# Quantitative MRI assessment of multifidus and erector spinae fatty degeneration in Parkinson's disease patients with lumbar degenerative disease: a propensity score-matched study

**DOI:** 10.3389/fmed.2026.1935592

**Published:** 2026-09-16

**Authors:** Ziming Li, Lei Wang, Jingming Wang, Xiaoduo Xu, Xin Chen, Yijie Kong, Xiaoyu Zhou, Weimin Huang

**Affiliations:** Department of Orthopaedics, The 960th Hospital of the PLA, Jinan, China

**Keywords:** erector spinae, lumbar degenerative disease, magnetic resonance imaging, multifidus, Parkinson disease, propensity score matching, semi-quantitative intramuscular fat-infiltration index

## Abstract

**Objective:**

This study aims to elucidate the characteristics of the multifidus and erector spinae muscles in patients suffering from lumbar degenerative diseases (LDD) complicated by Parkinson's disease (PD), through a comparative analysis with LDD patients who do not have PD.

**Methods:**

Seventeen patients with LDD accompanied by PD (case group) and 17 patients with LDD without PD (control group) were enrolled through propensity score matching (1:1) from January 2020 to January 2025. All participants underwent 1.5T lumbar MRI, and the semi-quantitative intramuscular fat-infiltration index (SIFI) and cross-sectional area (CSA) of the multifidus and erector spine muscles at the L3/4 and L4/5 levels were measured separately at each level.

**Results:**

The semi-quantitative intramuscular fat-infiltration index (SIFI) values of multifidus (66.19 ± 14.03) and erector spinae (73.54 ± 11.76) averaged across L3/4 and L4/5 level in the case group were significantly higher than those in the control group (56.63 ± 4.84 and 53.34 ± 5.90, all *P* < 0.05). However, no significant difference in the CSA of either muscle was observed between the two groups at either L3/4 or L4/5 level (*P* > 0.05). Additionally, the erector spinae exhibited greater fat infiltration than the multifidus in patients with PD, with no evidence of bilateral asymmetry or segmental predominance.

**Conclusion:**

Compared to LDD patients without PD, those with PD exhibit more severe fat infiltration in both the multifidus and erector spinae, primarily affecting the erector spinae. This fat infiltration compromises spinal stability, postural maintenance, and motor control, potentially contributing to spinal complications such as camptocormia. These findings suggest a potential therapeutic target for clinical intervention.

## 1 Introduction

Parkinson's disease (PD) is a prevalent neurodegenerative disorder characterized primarily by the degeneration and loss of dopaminergic neurons in the substantia nigra, which constitutes its core pathological feature (1, 2). The disorder is predominantly manifested through motor symptoms, such as bradykinesia, resting tremor, and muscular rigidity (3). Beyond these typical motor manifestations, patients with PD frequently experience a range of non-motor symptoms and musculoskeletal complications (4). Among these, low back pain, postural deformity, and an increased risk of falls are particularly prominent. These complications significantly compromise the quality of life for patients and elevate their rates of disability and mortality (5).

Muscle pathology represents one of the primary pathological foundations of motor dysfunction and musculoskeletal complications in patients with PD. Current research on muscle abnormalities in PD has predominantly concentrated on the skeletal muscles of the extremities, such as the quadriceps femoris and biceps brachii, which have been shown to exhibit alterations including muscle fiber atrophy, fat infiltration, and diminished strength (6, 7). However, the involvement of paraspinal muscles—anatomical structures essential for maintaining spinal stability—has not been adequately addressed in PD research. Among the paraspinal muscles, the multifidus and erector spinae serve as the core stabilizing and motive musculature of the spine, respectively, and the integrity of their function is crucial for determining spinal physiological curvature and motor stability (8). Limited clinical observations have indicated that PD patients may experience compromised paraspinal muscle strength and abnormal morphology (2); however, relevant studies primarily depend on subjective physical examinations or qualitative imaging descriptions, thus lacking objective quantitative analysis data derived from MRI.

This study employed MRI-based semi-quantitative analysis to determine the characteristics of the multifidus and erector spinae muscles in patients with PD complicated by lumbar degenerative disease (LDD). The aim was to clarify the pathological mechanisms underlying spine-related complications in these patients and to provide theoretical support for the development of targeted clinical intervention strategies.

## 2 Patients and methods

### 2.1 Study subjects

Seventeen patients with LDD who were also diagnosed with PD were enrolled in this study, Hoehn–Yahr stage 1–3, all of whom were admitted to the Department of Orthopedics at our hospital between January 2020 and January 2025. The sample size was determined by the number of patients meeting the stringent inclusion and exclusion criteria within this specified study period, rather than by *a priori* statistical power calculation. After 1:1 propensity score matching, 17 patients were included in each group.

The inclusion criteria were as follows: 1) fulfillment of the diagnostic criteria for PD established by the International Parkinson and Movement Disorder Society (MDS) in 2015 (9); 2) ability to cooperate with clinical treatment and undergo lumbar spine MRI examination; and 3) a disease duration of at least one year.

The exclusion criteria included: 1) comorbidities involving organic spinal diseases such as spinal trauma, tumors, or tuberculosis; and 2) comorbidities with other neuromuscular disorders such as muscular dystrophy or myasthenia gravis.

In parallel, 17 volunteers with LDD who underwent lumbar spine MRI at our hospital were selected as the control group through propensity score matching (PSM). The inclusion criteria for the control group were: 1) age and gender matched with the case group (age difference ≤3 years); 2) no history of PD or other neurological diseases; 3) no history of muscle disorders; and 4) absence of significant motor dysfunction.

Informed consent was obtained from all participants in both groups, and the study received approval from the Medical Ethics Committee of our hospital (see Figures 1, 2).

**Figure 1:**
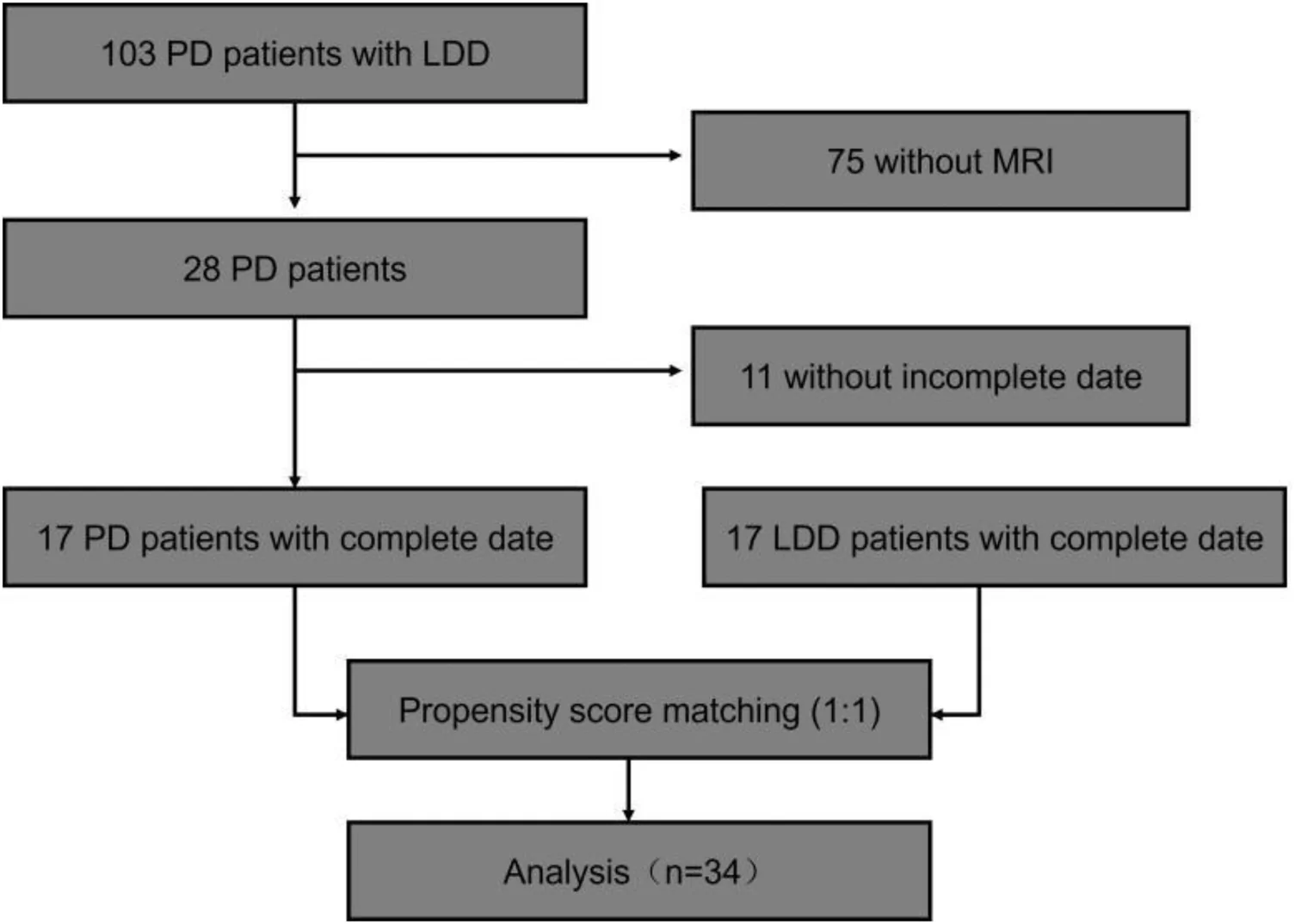
Patient inclusion and exclusion flowchart.

**Figure 2:**
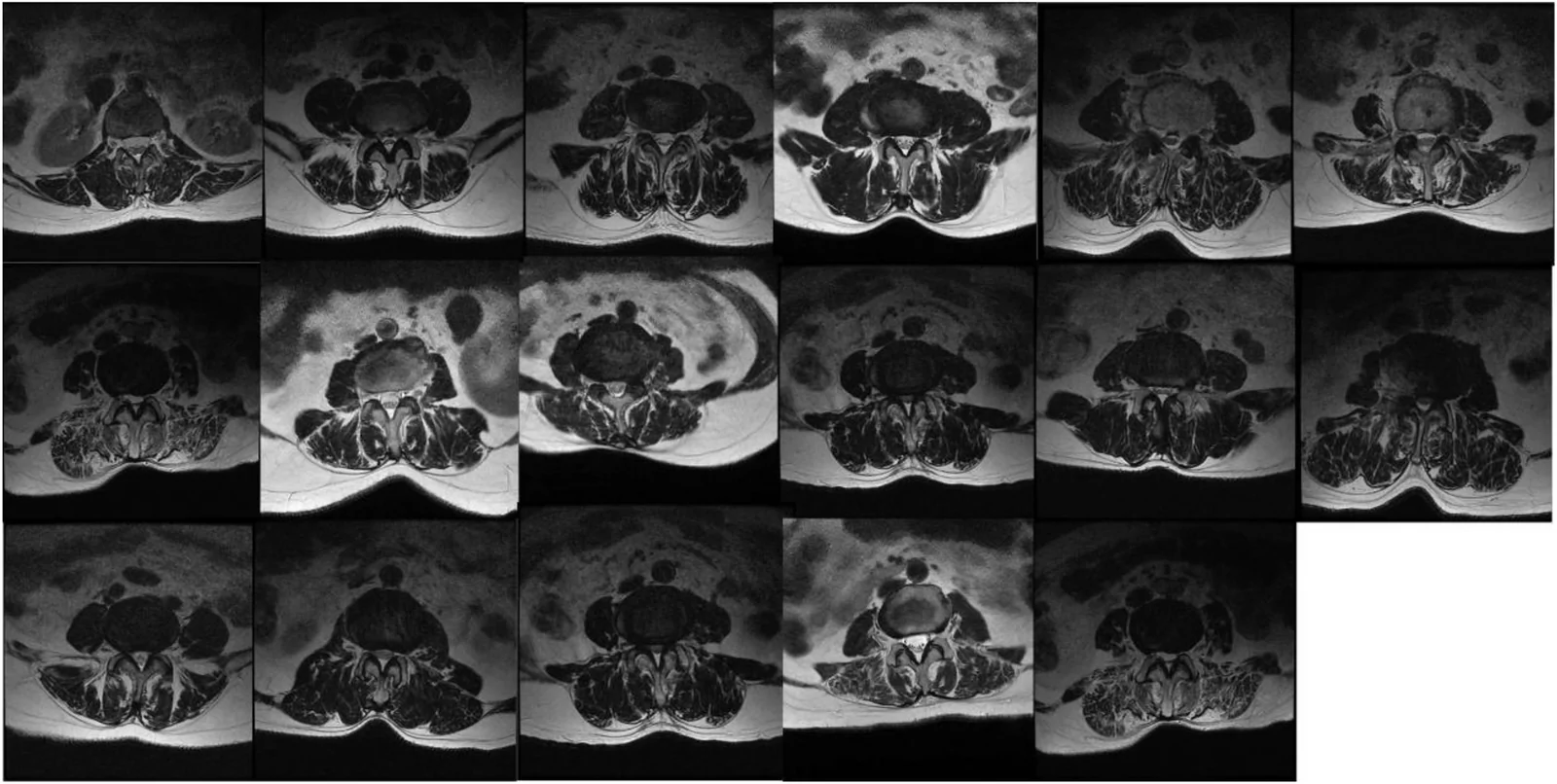
Representative cross-sectional MRI images of the multifidus and erector spinae muscles at both the L3/4 and L4/5 vertebral levels in patients with PD. (The images shown are representative sections from the L3/4 and L4/5 levels, respectively.)

### 2.2 Data collection

General clinical data for all subjects were collected, encompassing gender, age, height, and body weight, with the body mass index (BMI) calculated accordingly. Additionally, information regarding smoking history, alcohol consumption, and comorbid conditions, such as hypertension and diabetes, was documented. Data on patients' occupations and socioeconomic status were not systematically collected in this retrospective study, which we acknowledge as a limitation. For patients with PD in the case group, supplementary clinical data were gathered, including disease duration, types of anti-Parkinson medications administered (all patients received levodopa/benserazide), and disease severity was assessed using the Hoehn–Yahr staging scale.

### 2.3 MRI quantitative analysis

#### 2.3.1 Examination protocol

Lumbar spine MRI was conducted on all subjects utilizing a 1.5T scanner. The scanning sequences included T1-weighted imaging (T1WI), T2-weighted imaging (T2WI), and a fat suppression sequence. The scanning parameters were as follows: T1WI with a repetition time (TR) of 500 ms and an echo time (TE) of 15 ms; T2WI with a TR of 3,000 ms and a TE of 80 ms; slice thickness of 3 mm, interslice gap of 1 mm, a matrix of 256 × 256, and a field of view (FOV) of 30 cm × 30 cm.

#### 2.3.2 Image analysis

MRI images were independently analyzed by two senior orthopedic physicians using a double-blind method. In cases of discrepant opinions, a third senior physician was consulted, and a consensus was reached through collective discussion. The multifidus and erector spinae muscles at the L3/4 and L4/5 vertebral levels were selected as the regions of interest (ROIs). ImageJ image analysis software (National Institutes of Health, USA) was utilized to perform semi-quantitative measurement of paraspinal muscle intramuscular fat infiltration, defined as the semi-quantitative intramuscular fat-infiltration index (SIFI). This index was calculated as the ratio of fat-classified pixel area to total muscle-ROI area. Notably, this metric derived from conventional T1-weighted MRI threshold segmentation is semi-quantitative and cannot provide absolute volumetric fat fraction comparable to PDFF/Dixon-IDEAL MRI sequences. For threshold segmentation on each axial slice, signal statistics were sampled from subcutaneous fat within the same image; the segmentation cutoff threshold was set as mean signal intensity of subcutaneous fat minus 1.5 standard deviations. Within manually delineated ROIs of multifidus and erector spinae, pixels with signal intensity higher than this cutoff were categorized as fat-infiltrated regions. For each level (L3/4 and L4/5), SIFI and cross-sectional area (CSA) values were recorded separately for the left and right sides. Additionally, the bilateral symmetry of fat infiltration was recorded for all subjects (see Figure 3).

**Figure 3:**
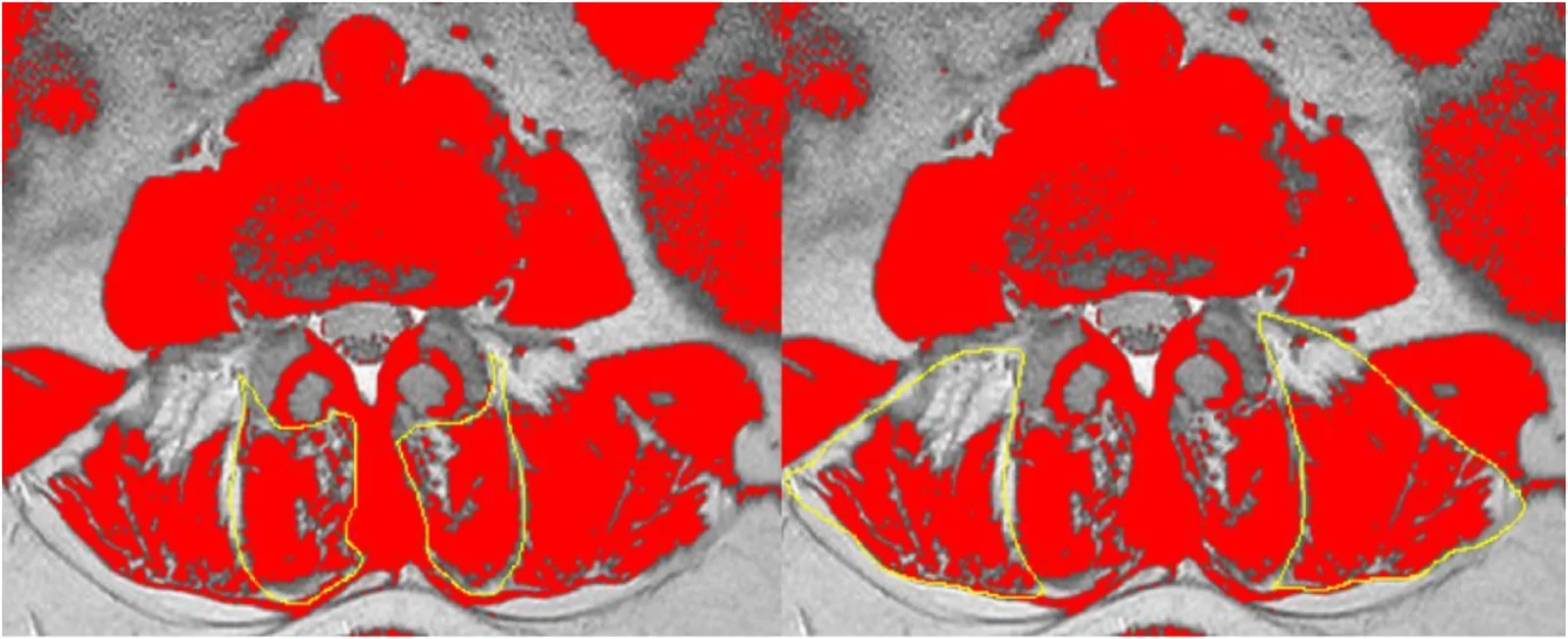
ImageJ was utilized to perform semi-quantitative measurement of paraspinal-muscle intramuscular fat-infiltration. The images display axial sections at the L3/4 (left) and L4/5 (right) levels. The multifidus and erector spinae are delineated by ROIs in yellow, respectively. The semi-quantitative intramuscular fat-infiltration index (SIFI) was calculated as the ratio of threshold-defined intramuscular adipose-like pixel area (hyperintense signal on T1WI) to total muscle area within each ROI. This measurement represents semi-quantitative fat-infiltration assessment rather than absolute volumetric fat-fraction. Muscle density was reflected by signal-intensity heterogeneity within the ROI.

### 2.4 Statistical analysis

All data were analyzed using SPSS version 26.0 (IBM Corp, USA). Measurement data were presented as mean ± standard deviation (x¯ ± s), and intergroup comparisons were conducted using the independent samples *t*-test. Categorical data were expressed as number (percentage) [n (%)], with intergroup comparisons performed using the chi-square test. Pearson correlation analysis was utilized to explore the relationships between the degree of paraspinal muscle fat infiltration and both severity and disease duration in patients with PD.

## 3 Results

### 3.1 Comparison of baseline data

The case group comprised 17 PD patients (9 males and 8 females), with a mean age of 65.2 ± 8.3 years, a mean disease duration of 5.3 ± 2.1 years, and a mean BMI of 23.5 ± 3.1 kg/m^2^. The control group consisted of 17 volunteers (9 males and 8 females) with a mean age of 64.8 ± 7.9 years. No statistically significant differences were observed between the two groups concerning gender, age, smoking history, alcohol consumption history, or the prevalence of underlying diseases (all *P* > 0.05), indicating adequate comparability. All PD patients underwent long-term treatment with levodopa/benserazide (see Table 1).

**Table 1:** Baseline characteristics between groups.

Index	Case group (*n* = 17)	Control group (*n* = 17)	*χ*^2^/*t* value	*P* value
Gender (male/female, *n*)	9/8	9/8	0.000	1.000
Age (years, x ± s)	65.2 ± 8.3	64.8 ± 7.9	0.144	0.886
BMI (kg/m^2^, x ± s)	23.5 ± 3.1	23.2 ± 2.8	0.321	0.750
Smoking history (yes/no, *n*)	7/10	5/12	0.725	0.395
Alcohol consumption history (yes/no, *n*)	6/11	5/12	0.208	0.649
Hypertension (yes/no, *n*)	13/4	14/3	0.217	0.641
Diabetes mellitus (yes/no, *n*)	4/13	3/14	0.152	0.697

BMI, body mass index.

### 3.2 Comparison of paraspinal muscle SIFI and cross-sectional area at L3/4 and L4/5

The SIFI values of the multifidus and erector spinae muscles in the case group were 66.19 ± 14.03 and 73.54 ± 11.76, respectively, which were significantly higher than those observed in the control group (*P* < 0.05). However, no statistically significant difference was found in the CSA of the paraspinal muscles between the two groups. The CSA of the multifidus and erector spinae muscles were 6,263.59 ± 2,082.63 mm^2^ and 7,408.06 ± 2,259.28 mm^2^ in the case group, compared to 5,308.71 ± 1,909.38 mm^2^ and 5,689.35 ± 1,974.52 mm^2^ in the control group, respectively (all *P* > 0.05) (See Table 2).

**Table 2:** Comparison of SIFI and CSA of the multifidus and erector spinae at L3/4 and L4/5 levels.

Level	Muscle	Parameter	Case group (*n* = 17)	Control group (*n* = 17)	*t*	*P*
L3/4	Multifidus	SIFI (%)	65.02 ± 13.87	56.21 ± 4.76	−2.512	0.018
		CSA (mm^2^)	6,189.34 ± 2,056.71	5,256.48 ± 1,887.23	−1.378	0.178
	Erector spinae	SIFI (%)	72.89 ± 11.52	52.97 ± 5.82	−6.198	<0.001
		CSA (mm^2^)	7,356.82 ± 2,241.15	5,634.19 ± 1,956.84	−0.872	0.390
L4/5	Multifidus	SIFI (%)	67.36 ± 14.19	57.05 ± 4.92	−2.802	0.009
		CSA (mm^2^)	6,337.84 ± 2,108.55	5,360.94 ± 1,931.53	−1.409	0.169
	Erector spinae	SIFI (%)	74.19 ± 12.00	53.71 ± 5.98	−6.457	<0.001
		CSA (mm^2^)	7,459.30 ± 2,277.41	5,744.51 ± 1,992.20	−0.901	0.375

SIFI, semi-quantitative intramuscular fat-infiltration index; CSA, cross-sectional area. SIFI derived from T1-weighted MRI threshold segmentation represents semi-quantitative measurement, not absolute volumetric fat fraction.

### 3.3 Imaging features of paraspinal muscle SIFI in PD patients

In the cohort of 17 PD patients, no statistically significant differences were observed in the SIFI values of the multifidus and erector spinae muscles between the right and left sides at the L3/4 and L4/5 vertebral levels (*P* > 0.05). Furthermore, no significant differences were identified in the SIFI values of the paraspinal muscles on the same side between the L3/4 and L4/5 levels (*P* > 0.05). These findings suggest a lack of pronounced segmental differences and bilateral asymmetry in paraspinal muscle fat infiltration among PD patients (see Table 3). Morphological analysis of MRI images indicated that, among the 17 PD patients, 12 cases (70.59%) exhibited patchy fat infiltration in the paraspinal muscles, while 5 cases (29.41%) displayed stellate fat infiltration. Bilateral symmetric fat infiltration was detected in 15 cases (88.24%), whereas asymmetric distribution was observed in only 2 cases (11.76%). Notably, all PD patients demonstrated paraspinal muscle fat infiltration across at least two vertebral levels.

**Table 3:** Imaging features of paraspinal muscle SIFI by side and level in PD patients.

Muscle group	L3/4 right	L3/4 left	L4/5 right	L4/5 left	F value	*P* value
Multifidus	58.24 ± 10.35	59.17 ± 11.26	56.39 ± 9.87	57.28 ± 10.52	0.215	0.886
Erector spinae	48.36 ± 9.78	47.52 ± 10.15	51.29 ± 11.34	52.17 ± 10.86	0.389	0.759

### 3.4 Correlation between SIFI and clinical characteristics of PD

The patients were divided into three subgroups according to Hoehn–Yahr (H–Y) staging: H–Y Ⅰ (*n* = 7), H–Y Ⅱ (*n* = 6), and H–Y Ⅲ (*n* = 4). Pearson correlation analysis showed that in the PD group, the SIFI values of the erector spinae and multifidus were positively correlated with disease duration (*r* = 0.621, *P* < 0.01; *r* = 0.538, *P* < 0.05) (See Table 4). With longer disease course, the paraspinal-muscle SIFI values gradually increased.

**Table 4:** Correlation between SIFI and clinical characteristics of PD.

Index	Multifidus SIFI		Erector spinae SIFI	
	*r*	*P*	*r*	*P*
Disease duration (years)	0.538	0.026	0.621	0.008
H–Y stage	4.127	0.035	6.892	0.007

## 4 Discussion

To the best of our knowledge, this study represents the first semi-quantitative investigation of the multifidus and erector spinae muscles in patients with LDD complicated by PD using MRI. Our primary findings indicate that, compared to patients with isolated LDD, those with PD exhibit significantly higher SIFI values in both the multifidus and erector spinae muscles, with the erector spinae showing a more pronounced degree of fat infiltration. In contrast, no significant differences were observed in the CSA of paraspinal muscles between the two groups. These findings suggest that the pathological effects of PD on the spine are characterized not by muscle atrophy *per se*, but by severe intramuscular fatty degeneration, particularly affecting the erector spinae. This pathological change may further compromise spinal stability (10–13), disrupt postural control, and contribute to the development of clinical sequelae such as Pisa syndrome or camptocormia (14, 12), which are frequently observed in advanced PD patients.

This finding suggests that the pathological alteration of paraspinal muscles in PD is not simple muscle fiber atrophy, but a process of muscle fiber replacement by adipose tissue. Adipose tissue infiltrates and proliferates in the muscle interstitium, filling the space left by degenerated muscle fibers. This results in no significant reduction in overall muscle volume but a marked decrease in the proportion of functional contractile muscle fibers, ultimately leading to impaired muscle strength.

Previous studies using ultrasound technology reported that the thickness of paraspinal muscles (e.g., the erector spinae) was significantly reduced in PD patients compared with healthy individuals (15–17), suggesting the presence of muscle atrophy. However, this finding contrasts with the results of our study, and this discrepancy warrants further discussion and comparison. Ultrasound technology has limitations in accurately distinguishing intramuscular adipose tissue from muscle fiber components, making it unable to achieve quantitative assessment of fat infiltration. In contrast, our study employed MRI-based semi-quantitative assessment of intramuscular fat-infiltration and confirmed that adult PD patients have a significantly higher prevalence of paraspinal muscle fat infiltration than healthy individuals, with the erector spinae being the most severely affected. This result identifies a critical pathological basis for spine-related complications in PD patients: paraspinal muscle fat infiltration leads to muscle atrophy and strength loss (18), impairs spinal stability, and further induces low back pain, postural deformity, and increased risks of falls and fractures (19, 20), thus providing a clear target for clinical intervention.

A noteworthy finding of our study is that the erector spinae exhibited more severe fat infiltration than the multifidus in PD patients, which differs from the pattern commonly observed in patients with purely mechanical LDD, where the multifidus is often reported to be more susceptible to fatty degeneration. We propose several explanations for this discrepancy. First, the erector spinae is a superficial muscle predominantly composed of type II (fast-twitch) glycolytic fibers, which are more vulnerable to neurogenic disuse and metabolic disturbances than the type I (slow-twitch) oxidative fibers that predominate in the multifidus. The dopaminergic denervation in PD leads to reduced voluntary activation of the erector spinae during postural adjustments, promoting selective disuse atrophy and subsequent fatty replacement. Second, the neural innervation patterns differ: the erector spinae is primarily innervated by the lateral branches of the posterior rami of spinal nerves, which have a more superficial course and are more susceptible to traction and compression from PD-related postural abnormalities (e.g., forward flexion and scoliosis). In contrast, the multifidus is innervated by the medial branches, which are deeper and relatively protected. Third, the anatomical space surrounding the erector spinae is comparatively loose, allowing easier infiltration of adipose tissue into the muscle interstitium once fiber atrophy occurs, whereas the multifidus is confined within a tight osseoligamentous compartment. Collectively, these factors—fiber type composition, neural vulnerability, and anatomical constraints—explain why the erector spinae, rather than the multifidus, emerges as a more sensitive imaging marker of PD-related paraspinal muscle degeneration. This finding has practical implications: SIFI of the erector spinae may serve as a more sensitive biomarker for early detection of spinal muscle involvement in PD and for monitoring disease progression.

The high incidence of fat infiltration in the erector spinae of PD patients may be associated with multiple mechanisms involving central neural innervation, anatomical location and muscle metabolism. At the central level, the core pathological feature of PD is the degeneration of dopaminergic neurons in the midbrain substantia nigra (21–23). Dopamine not only regulates limb movement but also participates in the motor coordination and energy metabolism of trunk muscles (24). The erector spinae is highly dependent on the central nervous system, particularly the dopaminergic pathway. Therefore, impairment of dopaminergic nerve function directly affects the neural innervation of the erector spinae (25), leading to attenuated muscle fiber anabolism and enhanced catabolism, which in turn induces muscle fiber atrophy and subsequent replacement by adipose tissue. In contrast, the neural innervation of the multifidus is not only regulated indirectly by the dopaminergic pathway but also predominantly dependent on intrinsic spinal neural circuits (e.g., the proprioceptive afferent-motor efferent reflex arc) and proprioceptive feedback (26). These pathways exhibit far less degeneration in the early stage of PD compared with the nigrostriatal pathway, resulting in minimal direct impact of central lesions on the neural innervation of the multifidus.

The more severe fat infiltration in the erector spinae compared with the multifidus may also be related to their distinct anatomical and functional characteristics. The erector spinae is a superficial paraspinal muscle mainly composed of type Ⅱ muscle fibers (fast-twitch fibers) and is responsible for driving large, voluntary spinal movements. In contrast, the multifidus is a deep paraspinal muscle predominantly composed of type Ⅰ muscle fibers (slow-twitch fibers) (27), which primarily maintains spinal segmental stability. Its neural innervation relies on the posterior rami of spinal nerves, and neurological lesions in PD may impair spinal nerve motor function, leading to preferential involvement of deep muscles. Furthermore, the differential fat infiltration may be associated with the anatomical location of the posterior rami of spinal nerves: the erector spinae is innervated by the lateral branches of the posterior rami, while the multifidus is innervated by the medial branches. The superficial anatomical location of the lateral branches renders them susceptible to muscle traction and fascial compression caused by long-term postural abnormalities (e.g., scoliosis and forward flexion) in PD patients, resulting in nerve conduction dysfunction. Impairment of the lateral branches leads to reduced motor coordination of the erector spinae, and the muscle undergoes accelerated fiber necrosis due to denervation combined with excessive compensatory contraction, which is subsequently replaced by adipose tissue. Additionally, the erector spinae, as a superficial paraspinal muscle, has a relatively loose anatomical space. When muscle fiber atrophy occurs, surrounding adipose tissue can easily infiltrate the muscle interstitium and replace the atrophic fibers, forming fat infiltration. In contrast, the multifidus is a deep paraspinal muscle located between the vertebral lamina and spinous process, with a narrow anatomical space and tight connections to spinal bones and ligaments, which hinders adipose tissue infiltration. In summary, the type Ⅱ muscle fibers of the erector spinae are more prone to atrophy and dysfunction in PD due to their dependence on dopaminergic regulation, anaerobic metabolism, and susceptibility to disuse. In contrast, the type Ⅰ muscle fibers of the multifidus are significantly less affected by PD due to their high endurance, autonomous metabolism, and continuous functional activation for spinal stability.

The disproportionate fat infiltration of paraspinal muscles in PD patients, independent of overt muscle atrophy, can be attributed to the unique pathophysiological interactions between neurodegeneration and musculoskeletal degeneration. First, chronic dopaminergic depletion in the nigrostriatal pathway disrupts the central motor regulation of paraspinal muscles, leading to persistent muscle hypotonia, disuse, and altered neuromuscular junction transmission. This chronic inactivity triggers the differentiation of muscle satellite cells into adipocytes instead of myofibers, resulting in selective fatty degeneration without significant loss of muscle volume. Second, PD-related systemic metabolic abnormalities, including mitochondrial dysfunction, oxidative stress, and chronic low-grade inflammation, create a microenvironment conducive to intramuscular fat deposition, exacerbating the degenerative process superimposed on pre-existing LDD. Unlike primary LDD, where paraspinal muscle degeneration is driven by mechanical stress and segmental instability, PD-related muscle damage is predominantly influenced by neurogenic-mediated metabolic remodeling, which explains the observed bilateral symmetry and lack of segmental specificity in fat infiltration. Furthermore, the erector spinae, as a tonic postural muscle with a high oxidative fiber content, is more vulnerable to neurogenic disuse and metabolic disturbances compared to the multifidus, which aligns with our finding of more severe fat infiltration in this muscle group.

Notably, the preserved CSA of paraspinal muscles in patients with PD challenges the conventional view that neurodegenerative myopathy is synonymous with muscle atrophy. We speculate that the mild compensatory hypertrophy of residual myofibers or interstitial edema may offset the volume loss caused by myofiber degeneration, thereby masking the underlying structural damage. This observation also suggests that SIFI is a more sensitive and early imaging indicator for evaluating paraspinal muscle impairment in PD patients compared to CSA, which is crucial for the early identification of spinal musculoskeletal complications.

Our findings have significant clinical implications for the diagnosis, risk stratification, and targeted management of PD patients with concurrent LDD. The quantitative assessment of paraspinal muscle SIFI via lumbar MRI provides an objective, non-invasive biomarker for spinal muscle degeneration, enabling the early detection of subclinical myopathy prior to the onset of severe postural deformity or refractory low back pain (28). This is a critical consideration, as PD patients often attribute such symptoms to primary lumbar degeneration, leading to a delayed recognition of neurogenic muscle damage. The identification of severe fat infiltration as a key pathological feature further underscores a novel therapeutic target for mitigating spinal complications in PD. While current management strategies primarily focus on anti-parkinsonian medications and conservative lumbar treatments, targeted interventions aimed at inhibiting intramuscular fat deposition and enhancing neuromuscular function—such as task-specific postural rehabilitation, neuromuscular electrical stimulation, or metabolic modulators—may improve spinal stability and alleviate motor symptoms. Notably, the symmetric pattern of fat infiltration may serve as an imaging biomarker to differentiate neurogenic lumbar dysfunction from mechanical LDD, thereby guiding personalized treatment strategies and avoiding unnecessary surgical interventions. These insights emphasize the necessity of bridging neurology and orthopedics through multidisciplinary collaboration in the management of PD. Routine MRI evaluations of paraspinal muscles should be considered for PD patients presenting with chronic low back pain or postural abnormalities to facilitate early interdisciplinary interventions and improve functional outcomes.

This study has several limitations. First, this is a single-center, retrospective study with a relatively small sample size (17 patients per group), which may limit statistical power and generalizability. The sample size was constrained by the number of patients meeting the strict inclusion criteria within the study period (January 2020 to January 2025) and the 1:1 propensity score matching process; therefore, multi-center, large-sample prospective studies are warranted to validate these findings. Second, we did not conduct long-term follow-up to assess dynamic changes in paraspinal muscle SIFI and their correlation with the progression of PD motor symptoms; longitudinal cohort designs are necessary to clarify the causal relationship between fat infiltration and disease progression. Third, baseline data on patients' occupations and socioeconomic status were not collected in this retrospective analysis; these factors may influence physical activity levels, access to healthcare, and disease management, and should be included in future prospective studies. Fourth, histological verification was not obtained due to the invasive nature of muscle biopsy in this patient population; however, MRI quantitative parameters have been widely validated as reliable surrogates for muscle histopathology. Fifth, the single-center design may introduce selection bias, and the generalizability of our findings to other populations or healthcare settings requires confirmation. Sixth, our assessment of muscle fat infiltration relied on semi-quantitative threshold segmentation of conventional T1-weighted MRI rather than gold-standard PDFF/Dixon-IDEAL sequences, which cannot deliver scanner-independent absolute volumetric fat-fraction values. Cross-literature direct numerical comparison is limited. Future investigations should incorporate dedicated quantitative fat-MRI protocols.

## 5 Conclusion

Compared to LDD patients without PD, those with PD exhibit more severe fat infiltration in the paraspinal muscles, primarily affecting the erector spinae. This fat infiltration compromises spinal stability, postural maintenance, and motor control, potentially contributing to spinal complications such as camptocormia. These findings suggest a potential therapeutic target for clinical intervention.

## Data Availability

The original contributions presented in the study are included in the article/Supplementary Material, further inquiries can be directed to the corresponding author.

## References

[1] Reich SG Savitt JM. Parkinson's disease. Med Clin North Am. (2019) 103(2):337–50. 10.1016/j.mcna.2018.10.014 30704685

[2] Sveinbjornsdottir S. The clinical symptoms of Parkinson's disease. J Neurochem. (2016). 139:318–24. 10.1111/jnc.13691 27401947

[3] Fukunaga JY Quitschal RM Doná F Ferraz HB Ganança MM Caovilla HH. Postural control in Parkinson's disease. Braz J Otorhinolaryngol. (2014) 80(6):508–14. 10.1016/j.bjorl.2014.05.032 25457071 PMC9442668

[4] Morimoto R Kitagawa K Todo K Iijima M. Clinical analysis of sarcopenia prevalence and its influencing factors in patients with Parkinson's disease. Front Aging Neurosci. (2025) 17:1718723. 10.3389/fnagi.2025.1718723 41439005 PMC12719491

[5] Hayes MT. Parkinson's disease and parkinsonism. Am J Med. (2019) 132(7):802–7. 10.1016/j.amjmed.2019.03.001 30890425

[6] Gamborg M Hvid LG Thrue C Johansson S Franzén E Dalgas U. Muscle strength and power in people with Parkinson disease: a systematic review and meta-analysis. J Neurol Phys Ther. (2022) 47(1):3–15. 10.1097/NPT.0000000000000421 36318503

[7] Barichella M Cereda E Ferri V Bolliri C Cereda V Colombo A. Sarcopenia, low muscle strength, cognitive functions, and quality of life in parkinsonian syndromes. Nutrition. (2024) 128:112568. 10.1016/j.nut.2024.112568 39305523

[8] Li Y Zhang X Xu X Xie S Sun C Dong J. The morphology and composition of paravertebral muscles evaluated by MRI technology are used to predict low back pain: a systematic review and meta-analysis. Acad Radiol. (2025) 32(8):4750–66. 10.1016/j.acra.2025.04.026 40348711

[9] Postuma RB Berg D Stern M Poewe W Olanow CW Oertel W. MDS clinical diagnostic criteria for Parkinson's disease. Mov Disord. (2015) 30(12):1591–601. 10.1002/mds.26424 26474316

[10] Wijemanne S Jankovic J. Hand, foot, and spine deformities in parkinsonian disorders. J Neural Transm (Vienna). (2019) 126(7):1–12. 10.1007/s00702-019-01986-1 30809710

[11] Choi HJ Smith JS Shaffrey CI Lafage VC Schwab FJ Ames CP. Coronal plane spinal malalignment and Parkinson's disease: prevalence and associations with disease severity. Spine J. (2014) 15(1):115–21. 10.1016/j.spinee.2014.07.004 25041726

[12] Doherty KM van de Warrenburg BP Peralta MC Silveira-Moriyama L Azulay JP Gershanik OS. Postural deformities in Parkinson's disease. Lancet Neurol. (2011) 10(6):538–49. 10.1016/S1474-4422(11)70067-9 21514890

[13] Baker JF McClelland S 3rd Hart RA Bess RS. Management of spinal conditions in patients with Parkinson disease. J Am Acad Orthop Surg. (2017) 25(8):e157–65. 10.5435/JAAOS-D-16-00627 28692583

[14] Barone P Santangelo G Amboni M Pellecchia MT Vitale C. Pisa syndrome in Parkinson's disease and parkinsonism: clinical features, pathophysiology, and treatment. Lancet Neurol. (2016) 15(10):1063–74. 10.1016/S1474-4422(16)30173-9 27571158

[15] Elliott-Burke T Dillon T Bailey J Miller S Joos R Buros Stein A. Lumbar multifidus muscle ultrasound imaging: is handheld technology reliable? Musculoskelet Sci Pract. (2023) 65:102771. 10.1016/j.msksp.2023.102771 37182391

[16] Masaki M Takeuchi M Kasahara M Minakawa K Inagaki Y Ogawa Y. Association of activities of daily living, mobility and balance ability, and symptoms of Parkinson's disease with the masses and amounts of intramuscular non-contractile tissue of the trunk and lower extremity muscles in patients with Parkinson's disease. J Med Ultrason (2001). (2023) 50(4). 10.1007/s10396-023-01356-1 37646863

[17] Masaki M Kasahara M Inagaki Y Yokota M Takeuchi M. Association of sagittal spinal alignment in the standing position with the masses and amounts of intramuscular non-contractile tissue of the trunk and lower extremity muscles in patients with Parkinson's disease. Clin Biomech (Bristol). (2022) 101. 10.1016/j.clinbiomech.2022.105868 36580828

[18] Ondo WG Haykal HA. Paraspinal muscle asymmetry in Parkinson's disease. Int J Neurosci. (2013) 124(2). 10.3109/00207454.2013.825259 23865391

[19] Yun YR Yeom JS Lee JS Kim D Lee YJ Ha IH. Integrative postural rehabilitation for kyphotic deformity in a patient with Parkinson's disease: a case report and literature review. J Clin Med. (2025) 14(11). 10.3390/jcm14113705 PMC12156823 40507466

[20] Xu HW Yi YY Li YZ Zhang SB Wang SJ Wu DS. Sagittal imbalance, muscle atrophy, and osteoporosis: risk factors for revision posterior lumbar fusion surgery in patients with Parkinson's disease. Int Orthop. (2020) 44(10). 10.1007/s00264-020-04666-y 32564174

[21] Popovic Z Gilman Kuric T Rajkovaca Latic I Matosa S Kusic L de Gobbis A. Smooth pursuit and memory saccades are impaired in early-stage Parkinson's disease patients. Front Neurol. (2026) 16. 10.3389/fneur.2025.1702050 41659980 PMC12873708

[22] Smajić S Prada-Medina CA Landoulsi Z Ghelfi J Delcambre S Dietrich C. Single-cell sequencing of human midbrain reveals glial activation and a Parkinson-specific neuronal state. Brain. (2021) 145(3). 10.1093/brain/awab446 PMC9050543 34919646

[23] Marino BLB de Souza LR Sousa KPA Ferreira JV Padilha EC da Silva CHTP. Parkinson's disease: a review from pathophysiology to treatment. Mini Rev Med Chem. (2019) 20(9). 10.2174/1389557519666191104110908 31686637

[24] Santos PCR Heimler B Koren O Flash T Plotnik M. Dopamine improves defective cortical and muscular connectivity during bilateral control of gait in Parkinson's disease. Commun Biol. (2024) 7(1):495. 10.1038/s42003-024-06195-5 PMC11043351 38658666

[25] Caiyong LCSX. Anatomical study of neck back muscle and spinal nerve dorsal rami. Chin J Neurosurg. (2014) 30(8):811–4. 10.3760/cma.j.issn.1001-2346.2014.08.016

[26] Qiao PL Tayier A. Change of paravertebral muscle in patients with lumbar degenerative disease. J Clin Rehabil Tissue Eng Res. (2014) 44:7205–10. 10.3969/j.issn.2095-4344.2014.44.028

[27] Cagnie B Dhooge F Schumacher C De Meulemeester K Petrovic M van Oosterwijck J. Fiber typing of the erector spinae and Multifidus muscles in healthy controls and back pain patients: a systematic literature review. J Manipulative Physiol Ther. (2015) 38(9):653–63. 10.1016/j.jmpt.2015.10.004 26547762

[28] Vandenborne K Walter GA Straub V Willcocks RJ Forbes SC Mercuri EM. Quantitative muscle magnetic resonance outcomes in patients with Duchenne muscular dystrophy: an exploratory analysis from the EMBARK randomized clinical trial. JAMA Neurol. (2025) 82(7):734–44. 10.1001/jamaneurol.2025.0992 40354061 PMC12070283

